# Dysfunctional High-Density Lipoprotein Cholesterol and Coronary Artery Disease: A Narrative Review

**DOI:** 10.3390/jpm14090996

**Published:** 2024-09-19

**Authors:** Cristina Madaudo, Giada Bono, Antonella Ortello, Giuseppe Astuti, Giulia Mingoia, Alfredo Ruggero Galassi, Vincenzo Sucato

**Affiliations:** Division of Cardiology, Department of Health Promotion, Mother and Child Care, Internal Medicine and Medical Specialties (ProMISE), University Hospital Paolo Giaccone, University of Palermo, 90127 Palermo, Italy

**Keywords:** coronary artery disease, dysfunctional HDL, residual cardiovascular risk, inflammation, endothelial dysfunction

## Abstract

High-density lipoprotein (HDL) cholesterol is traditionally viewed as protective against cardiovascular disease (CVD). However, emerging evidence reveals that dysfunctional HDL, characterized by impaired reverse cholesterol transport (RCT), reduced anti-inflammatory and antioxidant activities and increased endothelial dysfunction, which can contribute to coronary artery disease (CAD). Dysfunctional HDL, resulting from oxidative modifications of Apolipoprotein A-1 (Apo A-1) and enzyme inactivation, fails to effectively remove cholesterol from peripheral tissues and may promote inflammation and atherosclerosis. Genetic mutations affecting HDL metabolism further complicate its role in cardiovascular health. Studies have shown that conventional therapies aimed at raising HDL-C levels do not necessarily reduce cardiovascular events, highlighting the need for new approaches that improve HDL functionality. Therapeutic strategies such as Apo A-1 mimetic peptides, reconstituted HDL infusions, and drugs targeting specific HDL metabolic pathways are being explored. Additionally, weight loss, statin therapy, and niacin have shown potential in enhancing HDL function. The pathophysiology of dysfunctional HDL involves complex mechanisms, including oxidative stress, inflammation, and genetic mutations, which alter its structure and function, diminishing its cardioprotective effects. New functional assays, such as the cholesterol efflux capacity (CEC) and HDL inflammatory index, provide more accurate predictions of cardiovascular risk by assessing HDL quality rather than quantity. As research progresses, the focus is shifting towards therapeutic strategies that enhance HDL function and address the root causes of its dysfunction, offering a more effective approach to reducing cardiovascular risk and preventing CAD.

## 1. Introduction

High-density lipoprotein (HDL) cholesterol is traditionally recognized for its protective role in reducing the risk of cardiovascular disease (CVD). However, emerging evidence suggests that HDL could be dysfunctional, losing its traditional cardioprotective properties and potentially contributing to coronary artery disease (CAD). 

The quality and functionality of HDL, rather than its quantity, are now recognized as critical determinants in CAD risk.

**Figure 1:**
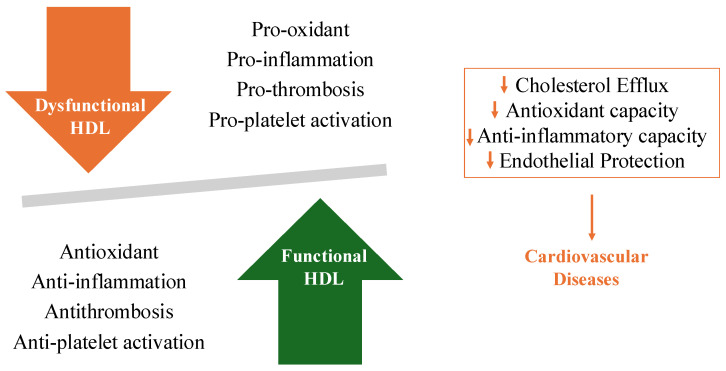
The characteristics of dysfunctional HDL and functional HDL.

Normally, reverse cholesterol transport (RCT) facilitates the efflux of cholesterol from peripheral tissues to the liver for excretion. Oxidative modifications of Apolipoprotein A1 (Apo A-1) and other structural changes can impair HDL’s interaction with cholesterol transporters such as the ATP-binding cassette transporter A1 (ABCA1) and ABCG1, thereby reducing its efficacy in RCT [1,2]. Then, dysfunctional HDL has a lower cholesterol efflux capacity (CEC), largely due to modifications in its primary protein component, Apo A-1, promoting the expression of vascular adhesion molecules and increasing monocyte adhesion, which exacerbates endothelial dysfunction and atherosclerosis [3,4]. Dysfunctional HDL often shows reduced activity of enzymes such as paraoxonase 1 (PON1) and glutathione peroxidase, resulting in increased oxidative stress and promotion of the formation of oxidized low-density lipoprotein (LDL), which are critical in atherogenesis [5,6]. Dysfunctional HDL, however, fails to stimulate nitric oxide (NO) production effectively, leading to endothelial dysfunction, which is a precursor to atherosclerosis [3,7]. Furthermore, mutations in the gene encoding scavenger receptors such as class B type I (SR-BI) [8,9], a receptor involved in HDL metabolism, have been linked to increased HDL cholesterol levels but also paradoxically to higher CAD risk, indicating the importance of HDL functionality over mere HDL-C levels [10]. The presence of dysfunctional HDL has significant clinical implications. Conventional therapies that aimed at raising HDL-C levels, such as the use of niacin or cholesteryl ester transfer protein (CETP) inhibitors, have not consistently led to reduced cardiovascular events, underscoring the need for therapeutic approaches that improve HDL functionality and target the underlying causes of HDL dysfunction, such as oxidative stress and inflammation [11]. Apo A-1 mimetic peptides and infusions of reconstituted HDL have shown promise in restoring HDL function and providing cardiovascular benefits [12,13]. The aim of this review is to address the role of HDL in preventing CVD and better define dysfunctional HDL, characterized by impaired RCT, reduced anti-inflammatory and antioxidant activities, and endothelial dysfunction that may contribute to CAD progression (Figure 1). Greater knowledge on the topic may guide future therapeutic strategies focusing on enhancing HDL function rather than simply increasing HDL-C levels to effectively reduce cardiovascular risk [14,15].

## 2. Materials and Methods

We conducted a comprehensive state-of-the-art literature search using databases such as PubMed, Scopus, and Google Scholar for articles in the literature from the last 20 years. Key terms included “dysfunctional HDL” OR “dysfunctional HDL and coronary artery disease”. Data extraction was performed by two independent reviewers (G.B., C.M.).

### Inclusion and Exclusion Criteria

Eligible studies were peer reviewed articles written in English. The last search was conducted in May 2024. Exclusion criteria included case reports, non-English publications, and studies with insufficient outcome data.

## 3. Roles of HDL in Protecting against CVD

HDL cholesterol has a central role in protecting against CVD through different mechanisms. HDL has different protecting mechanisms against CVD, including reverse cholesterol transport, anti-inflammatory, antioxidant, endothelial-protective, and antithrombotic effects. The focus on HDL functionality over HDL-C levels offers a more effective approach, exploiting the full protective potential of HDL. The traditional mechanism is RCT, which consists of the removal of excess cholesterol from peripheral tissues and its transport to the liver for excretion. This process is ensured by Apo A-1, a major protein component of HDL, which interacts with cholesterol transporters such as ABCA1 and ABCG1 on macrophages and allows for cholesterol efflux [2,16].

Furthermore, HDL has anti-inflammatory properties, inhibiting the expression of adhesion molecules and the production of pro-inflammatory cytokines, thus reducing monocyte recruitment to the endothelium and attenuating vascular inflammation. SR-BI modulates macrophage behavior, reducing the production of pro-inflammatory cytokines such as tumor necrosis factor-alpha (TNF-α) and interleukin-6 (IL-6) [8,17]. Then, different genetic studies have shown that variants in gene encoding proteins involved in HDL metabolism, such as SR-BI, have been associated with HDL dysfunction and increased risk of CVD [9]. These findings highlight the need for therapies that improve HDL quality rather than merely increasing HDL-C levels [10,11]. Another essential aspect is the antioxidant function of HDL-associated enzymes, such as PON1 and glutathione peroxidase, that prevents the oxidative modification of LDL and subsequent endothelial dysfunction and inflammation [4,5,6].

Through the activation of endothelial NO synthase (eNOS), HDL promotes NO production, a potent vasodilator and inhibitor of platelet aggregation. This is essential for maintaining endothelial health and preventing the development of atherosclerosis by promoting vascular relaxation and reducing blood pressure [7,18].

HDL-associated Apo A-1 and S1P modulate platelet function, preventing excessive platelet activation and aggregation, maintaining normal hemostasis and protecting against thrombotic events [18,19]. This is particularly relevant in the context of acute coronary syndromes (ACSs), in which thrombus formation can lead to myocardial infarction. Despite the well-documented protective roles of HDL, clinical trials that focus exclusively on therapies that increase levels of HDL have not clearly demonstrated reductions in cardiovascular events.

New therapeutic strategies are currently being studied, such as the use of Apo A-1 mimetic peptides, infusions of reconstituted HDL, drugs targeting specific pathways involved in HDL metabolism with the aim of improving HDL’s protective functions, and other therapies that facilitate HDL CEC and boost anti-inflammatory and antioxidant properties, thus improving coronary microcirculation [8,12].

Further research to improve HDL function and understand the genetic factors influencing it will be essential to develop effective HDL-targeted therapies to reduce cardiovascular risk [13].

## 4. Pathophysiology of Dysfunctional HDL

HDL is recognized for its protective role against atherosclerosis. However, in recent years, numerous studies have consolidated the idea that, under certain conditions, HDL can exert pro-atherogenic effects. The pathophysiology of dysfunctional HDL is multifactorial, and these factors, taken together, compromise the ability of HDL to perform its protective functions, thus contributing to the development and progression of atherosclerosis and CVD. This means that, depending on the surrounding microenvironment, HDL can undergo alterations in chemical conformation and biological activity, changing its antioxidant nature and its active role in RCT.

**Figure 2:**
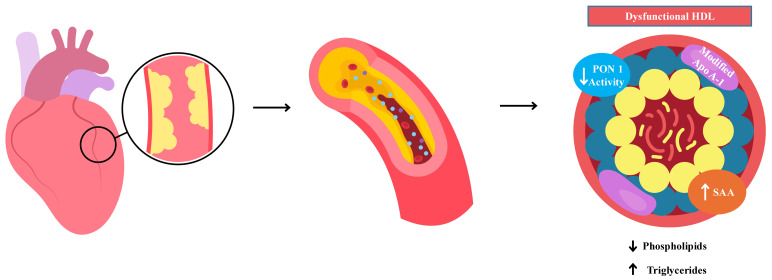
Modifications of selected HDL components in CVD pathophysiology.

RCT is a crucial process in the lipid homeostasis of peripheral cells, with HDL playing a key role. Specifically, HDL facilitates the efflux of cholesterol from cell membranes and its esterification by the enzyme LCAT. Cholesterol efflux from membranes depends on the lipid content in Apo A-1, which plays at least four fundamental roles (Figure 2):

- It is a structural component of HDL.
- It induces conformational changes necessary for the binding site between pre-β-HDL and membranes.
- It acts as a cofactor for LCAT enzyme activation.
- It is required for the selective uptake of esterified cholesterol into hepatocytes.

Some studies suggest that HDL may have other anti-atherogenic activities, such as antithrombotic effects [20], stabilizing activity on prostacyclin [21], the inhibition of adhesion molecule expression, and the release of factors for endothelial relaxation [22]. However, it is believed that one of the most significant contributions to the protective effect of HDL is due to the action of two enzymatic species associated with HDL: platelet-activating factor acetylhydrolase and serum paraoxonase, which can degrade the biologically active phospholipids of LDL.

Therefore, in addition to preventing cholesterol accumulation in the vascular wall, HDL protects against the development of atherosclerosis by inhibiting the oxidative modifications of plasma LDL. The pathophysiology of dysfunctional HDL involves several complex mechanisms that alter its structure and function [23]. A key factor in HDL dysfunction is oxidative stress. Oxidized HDL is the result of the enzymatic action of myeloperoxidase (MPO), which also induces the nitration and chlorination of Apo A-1. These modifications impair cholesterol efflux by altering the ABCA1, a crucial molecule in RCT [24]. Paraoxonase-1 (PON1), an enzyme associated with HDL that contributes to its antioxidant properties, can be inactivated in oxidative environments, reducing HDL’s ability to prevent the oxidation of LDL [5]. Reduced PON1 activity has been linked to an increased risk of CVD [5]. Inflammation also influences HDL activity. Some studies have shown that pro-inflammatory molecules can alter HDL conformation and function. These cytokines induce the expression of serum amyloid A (SAA), which can replace Apo A-1 on HDL, reducing its ability to promote cholesterol efflux (Figure 2) [7].

Inflammation can also stimulate the conversion of HDL to LDL by lipoprotein lipase [25]. LCAT is the enzyme responsible for cholesterol esterification and plays a crucial role in maintaining lipid homeostasis. Mutations in this enzyme, as well as in Apo A-1, ABCA1, and ABCG1, can lead to structural abnormalities in HDL particles. For example, LCAT deficiency results in the formation of small, dense HDL particles that are less effective in cholesterol efflux and antioxidant functions [26].

Emerging studies show the presence of dysfunctional HDL in various settings. South Asian patients with ACSs have elevated remnant cholesterol levels and an increased monocyte/HDL cholesterol ratio, indicating a higher inflammatory burden and altered lipid metabolism associated with dysfunctional HDL [27]. Furthermore, it is crucial to assess remnant cholesterol, the monocyte/HDL ratio, and lipoprotein ratios to personalize cardiovascular prevention strategies, addressing residual cardiovascular risk [28].

## 5. Role of Dysfunctional HDL on CAD

One of the paradigms acquired after the Framingham Heart Study and other epidemiological studies is the inverse correlation between HDL levels and cardiovascular risk. The antioxidant and anti-inflammatory properties of these molecules are well known, as is their primary function of recovering excess free cholesterol from macrophages and various peripheral tissue cells and transporting it to the liver, where it can be excreted via bile [29]. Glomset et al. were among the first to formulate the concept of RCT and suggest the involvement of HDL in protecting against atherosclerotic coronary disease [30]. Surprisingly, however, trials attempting to demonstrate the beneficial effects of therapies that increase HDL levels, such as CETP inhibitors, have only produced negative results, even leading to an increase in cardiovascular events [31,32]. Some authors have hypothesized that this could be due to CETP inhibition failing to protect HDL from oxidative stress, causing their dysfunction [33]. Further considerations arise from studies suggesting a U-shaped correlation between HDL cholesterol levels and mortality and comorbidity associated with CVDs, indicating that both very low and extremely high values are associated with an increased risk of cardiovascular mortality [34]. Moreover, certain genetic disorders leading to high HDL levels, do not necessarily correlate with reduced cardiovascular risk, as some mutations can cause a loss of function, resulting in the overproduction of abnormal HDL. It is also important to note that HDL is not homogeneous but represented by subpopulations with different compositions and sizes, which can confer different properties to these molecules [29].

HDL function, rather than cholesterol content, may be more important in cardiovascular disease. In a recent study, both low and high HDL-C levels were associated with increased mortality risk, so 50–79 mg/dL is recommended as the optimal HDL-C range among Chinese adults [35].

This understanding challenges the previous axiom based merely on the quantitative aspect of HDL and has shifted attention to the qualitative aspect. Dysfunctional HDL can not only fail to reduce cardiovascular events but may even increase atherosclerosis-related mortality if these particles acquire pro-inflammatory and pro-oxidant properties. While measuring HDL levels is simpler, evaluating their functionality through tests offers a more accurate prediction of cardiovascular risk. Recent guidelines suggest not using HDL levels as a risk predictor if plasma levels exceed 90 mg/dL (2.3 mmol/L) [36]. One functional test available is the CEC assay. Normally, mature HDL promotes cholesterol transport from macrophages and other cells through mediators such as ABCA1, ABCG1, and SR-BI. However, pro-inflammatory myeloperoxidases (MPOs) can cause oxidative stress, leading to HDL dysfunction, disabling transport through ABCA1 on macrophages and activating pro-inflammatory pathways. Khera’s group was among the first to identify the inverse relationship between atherosclerotic CAD and CEC, although further studies suggest that systemic inflammatory states with high C-reactive protein levels can reduce the predictive value of this test [37,38]. Another assay that provides information on HDL functionality is the HDL inflammatory index, which measures monocyte chemotactic activity (MCA) levels and the degree of LDL oxidation with a cell-free assay (CFA). The presence of HDL molecules with pro-oxidant and pro-inflammatory properties correlates with high MCA and CFA values, while the opposite occurs when HDL maintains its natural function [29].

Several enzymes and proteins contribute to proper HDL functionality, including PON1, which removes cholesterol from macrophages and prevents LDL particle oxidation, accounting for most of HDL’s antioxidant effect. Conversely, molecules such as myeloperoxidases can cause oxidative stress and impair the functions of Apo A-1. Some studies have evaluated the predictive role of measuring the plasma levels of these enzymes and particularly their ratio (MPO/PON1), as it appears evident that an imbalance favoring pro-oxidant activity can increase the risk of developing atherosclerotic diseases [39,40,41].

Among other molecules impacting HDL functionality worth mentioning are certain reactive carbonyl species, including malondialdehydes (MDAs) and isolevuglandins (IsoLGs), which interact with HDL, making it pro-inflammatory and pro-oxidant, thus losing its ability to promote RCT. Identifying these molecules has spurred interest in recognizing them as new therapeutic targets: an animal model study demonstrated that treatment with dicarbonyl scavengers, particularly 2-hydroxybenzylamine (2-HOBA), significantly reduced the extent of atherosclerotic plaques, stabilizing the fibrous cap and reducing the necrotic core area [42]. The fact that this result was achieved without altering the plasma levels of HDL-C, LDL-C, triglycerides, or other lipids further supports the impact that dysfunctional HDL can have on atherosclerotic disease and consequently on cardiovascular events. Currently, phase 1 human studies with 2-HOBA have demonstrated its safety, and phase 2 trials are underway in individuals with familial hypercholesterolemia to examine the effect of 2-HOBA on HDL modification and HDL CEC [43,44].

## 6. Treatment of Dysfunctional HDL

HDL function can be improved by weight loss achieved through diet or bariatric surgery and the use of statins, niacin, and CETP inhibitors [45].

Baynham et al. demonstrated that after a meal containing predominantly saturated fat, vascular inflammation persistently increased by overexpressing intercellular adhesion molecule 1 (ICAM-1) and vascular cell adhesion molecule 1 (VCAM-1) in human endothelial cells [46]. The diet, however, reduced monocyte chemotaxis in 22 patients with metabolic syndrome [47]. Roux-en-Y gastric bypass surgery, evaluated in 34 severely obese women, confirmed that weight loss was associated with an increase in HDL levels, as it enhanced the cholesterol efflux capacity (CEC) of macrophages through the SCARB1 and ABCG1 pathways [48]. Statin therapy further amplifies the effects of HDL on cholesterol efflux. In patients with type IIb hyperlipoproteinemia, atorvastatin treatment led to a dose-dependent increase in cholesterol efflux from cells [49]. On the other hand, statins might hinder the cholesterol efflux process mediated by HDL in macrophages via ABCA1-related mechanisms [50]. Studies in THP-1 cells have shown that statins raised miR-33 levels, leading to the suppression of ABCA1 expression, particularly with simvastatin. However, miR-33 does not impact the expression of ABCG1 or SCARB1 in human cells, meaning those efflux pathways mediated by HDL would remain functional. Moreover, it remains unclear whether the effects of statins observed in vitro are also relevant in vivo [50]. The Statin HDL Improvement in Function Trial (SHIFT), carried out in atherosclerosis patients who had not previously taken lipid-lowering drugs, found that simvastatin treatment improved the baseline inflammatory index. Nevertheless, PON activity was unaffected by the treatment, preventing full normalization of the inflammatory index [51]. The effects of niacin on different HDL functions are mixed. While niacin raises HDL-C levels and lowers triglycerides and apoB-containing lipoproteins, it only modestly enhanced cholesterol efflux from THP-1 macrophages loaded with cholesterol but had no effect on the efflux from J774 macrophages in statin-treated patients [52,53]. Consequently, niacin might not offer the same protective cardiovascular effects in statin-treated patients as it does when used as a standalone therapy [54,55]. In mice, niacin also lessens acute vascular inflammation and improves endothelial function [56]. In patients with type 2 diabetes, a 2 g daily dose of niacin for 2 months restored HDL’s ability to enhance endothelial NO production, decrease oxidative stress, and support endothelial repair [57].

Despite these findings, the randomized AIM-HIGH trial showed that adding niacin to statin therapy did not further reduce cardiovascular disease (CVD) risk in patients with low HDL-C and established atherosclerosis [12]. The HPS2-THRIVE study similarly found no additional reduction in CVD risk when niacin–laropiprant therapy was added to statin-treated patients with low LDL, non-HDL levels, and normal HDL levels [58]. CETP inhibitors cause a significant rise in HDL-C levels, but the first clinical trial with the CETP inhibitor torcetrapib was terminated due to off-target toxicity concerns [32]. Dalcetrapib, a weaker CETP inhibitor, raised HDL-C levels by 30–35% and increased HDL particle counts by 9%. However, the phase III dal-OUTCOMES trial in patients with prior ACS was discontinued due to minimal efficacy [31,59]. In vitro, the improved macrophage efflux stimulated by HDL under dalcetrapib treatment was reversed when co-incubated with a statin [49]. In the dal-ACUTE study, researchers explored the effects of dalcetrapib on HDL function markers within one week of an ACS event [60]. After 4 weeks of dalcetrapib treatment, the total cholesterol efflux capacity (CEC) increased by 9.5% compared to placebo, mostly driven by non-ABCA1-mediated transport. Anacetrapib and evacetrapib are newer CETP inhibitors that significantly raise HDL-C and reduce LDL-C levels [61,62]. Anacetrapib notably increased HDL-mediated macrophage cholesterol efflux via the ABCG1 pathway, and HDL from individuals treated with anacetrapib reduced macrophage inflammatory responses to Toll-like receptor 4 activation, like the effects observed with niacin treatment [52].

## 7. Conclusions

Recent research has highlighted the critical importance of HDL functionality over its quantity in protecting CVD. Dysfunctional HDL, characterized by reduced CEC, increased oxidative stress, and endothelial dysfunction can contribute to the progression of CAD. This shifts the focus towards therapies aimed at improving HDL quality and addressing the underlying causes of its dysfunction, such as oxidative stress and inflammation. Future therapeutic strategies should prioritize enhancing HDL’s protective functions to effectively reduce cardiovascular risk.

## Data Availability

Not applicable.
